# Legacies of humanitarian neglect: long term experiences of children who returned from the Lord’s Resistance Army in Uganda

**DOI:** 10.1186/s13031-021-00374-5

**Published:** 2021-05-29

**Authors:** Melissa Parker, Cristin A. Fergus, Charlotte Brown, Dorothy Atim, James Ocitti, Jackline Atingo, Tim Allen

**Affiliations:** 1 Department of Global Health and Development, London School of Hygiene & Tropical Medicine, London, UK; 2 Firoz Lalji Centre for Africa, London School of Economics and Political Science, London, UK; 3 Firoz Lalji Centre for Africa, Gulu, Uganda

**Keywords:** Return, Reintegration, Forced displacement, Post-conflict, Child soldiers, LRA, Uganda

## Abstract

**Background:**

Much has been written about the short-term challenges facing children returning ‘home’ from rebel fighting groups, but little is known about the longer term day to day realities of return. This article presents findings from the first long-term assessment of the social and economic challenges facing an officially registered group of children who passed through an internationally-financed reception centre after a period of time with the Lord’s Resistance Army (LRA).

**Methods:**

Records from a reception centre were used to trace a random sample of individuals to their current location. Two hundred and thirty in-depth semi-structured interviews were carried out and 40 follow-up interviews between 2013 and 2016**.** Interviews were informed by long-term ethnographic research in the region. These interviews were subsequently coded and analysed to describe the long-term day to day realities of return.

**Results:**

At the time of interview, 90% of formerly abducted people returned ‘home’ six or more years ago, and 75% returned nine or more years ago. The majority have managed to access family land for farming, but concerns about what they may have done to survive whilst living with the LRA adversely affects their day-to-day lives. However, some important differences were noted: those men and women who spent less time with the LRA are more likely to live on ancestral land with close relatives; and they are more likely to report experiencing stigma and a spiritual affliction called ‘cen’. In contrast, those who spent the longest time with the LRA are less likely to report these problems, they are mainly living in urban locations and tend to manage slightly better. Children born of war are vulnerable to abuse, irrespective of current residence.

**Conclusions:**

Research findings question the merits of post-conflict reintegration programmes emphasising immediate family reunifications, without follow-up monitoring, social protection, education and skills training. By overlooking the diverse experiences of those who lived and fought with the LRA, and failing to anticipate or respond to the long term socio-political and economic challenges facing children on their return, reception centre processes not only failed to foster social reintegration, but they also inadvertently exacerbated the vulnerability of returning children.

**Supplementary Information:**

The online version contains supplementary material available at 10.1186/s13031-021-00374-5.

## Introduction

Support for disarmament, demobilization and reintegration (DDR) of former combatants following protracted war and conflict occurs in the expectation that it will promote peace, national reconciliation and economic development [1]. In many instances, those passing through DDR processes are very young, and in some situations a considerable number may be children. Several scholars have attempted to assess what has actually happened to these people – most notably in Central and East Africa (for example, [2, 3]), Southern Africa (for example, [4–6]), West Africa (for example, [7–12]) and Central and South America (for example, [13–16]). Much of the research focuses on the social, psychological and economic challenges facing former recruits at the time of return, or soon afterwards. Only a few studies have attempted to document longer-term experiences. With respect to children passing through formal or informal DDR processes in Africa, hardly any such research has occurred. Important exceptions include work by Boothby [17, 18] Jordans et al. [19], and Betancourt et al. [20, 21]. Some points from these studies are worth noting at the outset.

Boothby [17], followed up a non-random sample of 39 male ex-child soldiers 16 years after their return. The children concerned spent anything from 2 months to 3 years fighting with Renamo in Mozambique, and a further 6 months at a rehabilitation centre, before being placed back with their families in the wider community. Importantly, the children were not just left with their relatives. They were followed up for 2 years. To facilitate their reintegration, children were encouraged to participate in apprenticeships and income-generating projects involving the provision of seeds and tools. These programmes took place alongside ‘community sensitisation programmes’, ‘traditional’ healing ceremonies and infrastructure projects supporting the welfare of all children (such as the re-building of hospitals). Although they all remain deeply affected by their experiences, the majority felt cared for and respected, by their families, friends and neighbours. Remarkably, this particular group were reported to be doing as well as, if not better, than children of a similar socio-economic status who had not perpetrated or witnessed extreme violence and murder as a child soldier.

Jordans et al. [19] carried out a larger study in Burundi, with 452 former child soldiers who had participated in an economic support programme four or more years after their return from combat. Their lives were compared with 191 peers who had not been recruited, but also participated in the programme. Funded by the International Labour Organisation, they all received skills training in brick-making, soap making, tailoring etc. as well as equipment kits and financial support. Again, the findings indicated that the two groups were ‘surprisingly similar in socio-economic functioning’ (2012:7). It is important to note, however, that the researchers relied on people self-reporting as former child soldiers and this may have affected the results, especially if study participants thought they had to say they had been a child soldier to secure a place on the economic support programme. Nevertheless, the relative success of the training programmes seems to have been significant.

Finally, Betancourt et al. [20, 21] documented the impact of war among 260 former child soldiers from Sierra Leone over a period of 6 years. By focusing on the way in which post-conflict factors (such as the nature and extent of community acceptance) influenced the mental health of returning combatants, their research suggested that, despite enduring distress associated with killing/injuring others during war and post-conflict stigma, some aspects of this distress could be mitigated by social support in schools and the wider community. A strong case is made for support programmes to recognise how wider social and economic issues shape trajectories of return. Research is on-going [22] and is usefully focusing on the intergenerational impacts of war for mental health and well-being.

In summary, while methods, sampling and focus make findings from this literature tentative, points emerging from these studies indicate that: follow up after reunification with families was important; effective skills training was an asset; assistance to communities hosting the returned children was helpful, and enduring distress can potentially be managed in a caring context. The present article adds to this small literature by presenting findings from a random sample of children who passed through an internationally-funded reception centre on their return from the Lord’s Resistance Army (LRA) in Uganda, with the vast majority having returned six or more years before their first interview.

### Assessments of return and reintegration in northern Uganda

It is striking that considerable interest was expressed in the challenges facing former recruits in the latter stages of the LRA war, and soon after hostilities subsided. Research activities proliferated, and this resulted in numerous publications (for example, [23–37]). Research documenting longer term processes is, however, limited. Where it has occurred, the authors are rather vague about the amount of time that has passed since the return of former recruits, and it is hard to gauge the generalisability of the issues being reported. This research is either based on ethnographic fieldwork with an ad hoc group (for example, [38–41]) or it is based on qualitative research involving men and/or women identified through NGOs, churches, schools and/or snowball sampling (for example, [42–44]). From a methodological point of view, it is fair to say that while long term ethnographic fieldwork provides fine-grained nuanced accounts of the social dimensions of return while simultaneously raising important ontological questions about the nature of trauma, well-being and identity, the reported findings are inevitably based on fieldwork with small numbers of people. Consequently, it is hard to gauge the generalisability of the issues being reported.

Slightly different methodological issues have arisen with qualitative social research involving former abductees identified through NGO’s, churches or snowball sampling. Here, there is a tendency for the research to take place in urban and peri-urban settings (where the NGOs and churches are located) and to rely on young people to come forward and self-identify as a formerly abducted person. Important issues about the social dimensions of return and reintegration have emerged from this research (such as the challenges facing women returning with children born of war), but it is far from clear how these findings relate to the experiences of those formerly abducted people who now live with their relatives in rural areas. A further limitation is that life in urban and peri-urban northern Uganda is precarious and many of the programmes run by NGOs (such as the provision of school fees for children born of war or training programmes in tailoring) end up assisting people who say they were abducted, but may actually have no history of abduction and need assistance for other reasons. In other words, the material incentives offered by NGOs has inadvertently encouraged people to present themselves as abductees, and to participate in research about abductees, even though they have not necessarily been abducted.

There is, however, one study which has attempted to document post-conflict social and economic challenges among 1844 randomly selected households between 2013 and 2015 [45]. To supplement this study, Atim et al. [46] carried out 57 interviews with women who had returned from the LRA, the majority of whom (63%) returned with a child. These interviews suggest that women returning with children born of war continue to face considerably more stigma than those women who did not return with a child. However, none of these women were selected randomly and no mention is made of the number of months and years that had passed since their return. It is, therefore, hard to know how these findings relate to the larger body of survey data collected.

Methodological limitations aside, there is a tendency to consider those returning from the LRA as very similar to each other. While some scholars have usefully foregrounded the way in which gender shapes experiences of return and reintegration [29, 46], little is known about variations between men, or between women, other than an emerging literature suggesting that women returning from the LRA with children born of war are particularly vulnerable and marginalised [46–48]. A temporal dimension documenting how experiences may have changed over time for men and women is also lacking. The findings presented below differ from this literature in that they offer an assessment of the long-term social and economic challenges facing children and young adults returning from life with the LRA, all of whom were randomly selected from official records from one reception centre.

#### The Ugandan context

The conflict involving the LRA under the leadership of Joseph Kony began in the mid-1980s. At the peak of the violence in the early 2000s, there were more than a million people living in internal displacement camps, most of whom came from Kony’s own Acholi ethnic group. The LRA frequently attacked the camps and perpetrated atrocities. In 2004, the situation was referred to the International Criminal Court, and in 2005, arrest warrants were issued for Kony and his senior commanders. Fighting continued until 2006, when the LRA were drawn into peace negotiations. These negotiations failed in 2008 and LRA activities have subsequently occurred in South Sudan, Democratic Republic of Congo and Central African Republic.

One of the LRA’s tactics is forced recruitment, or abduction. In northern Uganda, it is estimated that over 50,000 people were taken in this way, initially from their homes, and later from the internal displacement camps, which were not well defended by the Ugandan army [32, 49] Around half of those recruited were children (under 18 years), both males and females. The females were mostly given to LRA commanders as wives. The males and many of the females, were compelled to become combatants. To survive, many had to kill their own friends and relatives.

During the fighting, a large number of abducted children escaped, surrendered or were captured by the Ugandan army, and the army subsequently took responsibility for returning them to their families. They tried to do this by announcing their names over the radio and parading them in towns. Relatives were encouraged to come forward and collect their children, but they often failed to do so. Critical of these early attempts, a group of concerned parents established a reception centre called Gulu Support the Children Organisation (GUSCO) in 1997. Their intention was to facilitate the return of formally abducted people in a more caring and appropriate way.

Soon afterwards, World Vision opened another centre in Gulu town. A dozen more were established in the years that followed in other urban centres, and they all received international funding. The numbers returning were initially small (a few hundred), but once the Ugandan army crossed the border into Sudan (an area now referred to as South Sudan) in 2002, the numbers increased rapidly to around 6000 per year. It is estimated that the reception centres received around 25,000 children and young adults by 2006 [31]. Other adults also returned without going through the centres, reporting to the army and securing amnesty certificates.

At the reception centres, those returning from the LRA were provided with medical and nutritional care. In accordance with the UNICEF best practice guidelines [50–52], every effort was made to reunite them with their families – the majority of whom were living in internal displacement camps. Psychosocial assistance was also offered, but it was not delivered by trained therapists. It mainly involved providing clear instructions about appropriate ways to behave. Returnees were told to set aside aggressive ‘bush mentalities’ and encouraged to ignore anyone who spoke to them about their experiences in a negative and hostile way. Not only were they encouraged to think of themselves as ‘innocent victims’ who had been pressurised to behave in violent and murderous ways, but they were also told that their families and relatives would be forgiving and welcome them back home [23].

Little effort was made to check up on children and young adults once they had been placed back with their relatives. In part, this reflected the volatile security situation: permission to visit internal displacement camps was usually only granted under the condition of a nightfall curfew and a military escort. Additionally, movement in and out of the area was not easy because of the large number of Ugandan army road blocks, widespread (and justifiable) fears of land mines, and LRA attacks on the roads. Nevertheless, when the war ceased, no attempt was made by staff at internationally-funded reception centres to systematically find out what had happened to those children they had placed back ‘home’.

## Methods

To describe the long term experiences of these children (most of whom were young adults at the time of research), an inter-disciplinary study design was employed. This involved drawing upon historically-informed ethnographic research to inform both the design and interpretation of cross-sectional data. The sample was comprised of individuals who were less than 18 years old when they returned from the LRA and passed through GUSCO – a reception centre in Gulu town.

### Tracing individuals from GUSCO records

The sample was created in 2013, 7 years after the cessation of LRA attacks in northern Uganda. It was generated from a random selection of original reception centre records at GUSCO. Despite the decay and destruction of many records, the files of 3040 individuals registered at the centre between 1997 and 2012 survived. Based on previous work at the centre [23], an estimated 100 records were either missing or damaged beyond repair. Ten percent of the remaining records were selected (*n* = 304), and intensive efforts were made to find, and interview, the selected individuals.

It was not straightforward tracing individuals from the GUSCO records. In seven cases, it was found that the names in the reception centre registers were incorrect. In a further seventeen cases, individuals had changed their names when they returned to their relatives. Changing names served a dual purpose: it reduced the possibility of being re-abducted by the LRA while simultaneously enabling the person concerned to surreptitiously ‘fit back in’ to daily life. There were particular difficulties tracing women: typically, Acholi women move to their male partner/husband’s home when they marry. If problems emerge in the relationship, they return to their father’s homestead and, quite possibly, remarry. Re-marriage is high (as it is among women who do not have a history of abduction); and finding women, many of whom lived in places that were a considerable distance from their parental home, was not straightforward. Tracing men presented different challenges. Many were concerned that members of the research team might be government officials in disguise, and they suspected that they had a hidden agenda of recruiting them to the Ugandan army. It was thus not unusual for family members to say that the young man was not around, even if he was actually in the vicinity. Despite these challenges, 230 individuals were successfully traced, accounting for 75% of the sample originally selected.

**Fig. 1:**
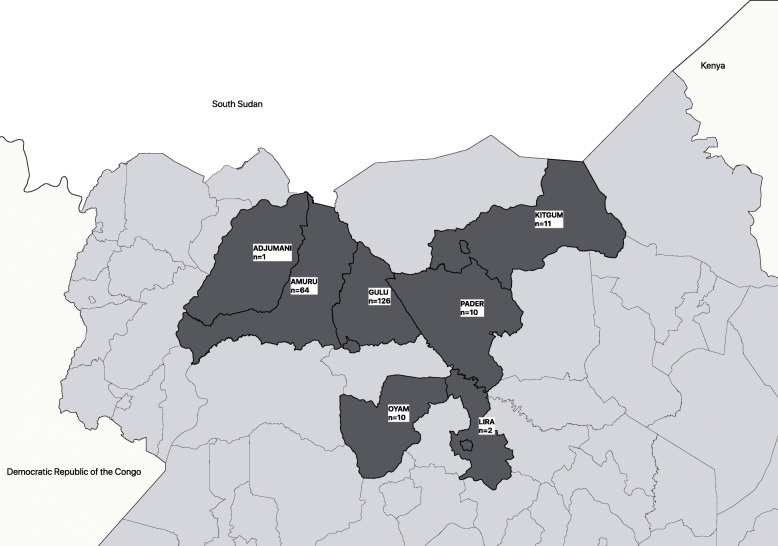
Map of districts indicating the locations where individuals were traced

The number of records sampled, and the number subsequently traced is shown in Table 1. The table presents this information for three time periods representing different phases of the conflict: 1997–2001, 2002–2005, and 2006–2016. The first time period refers to the period before the Ugandan army began attacking LRA bases in South Sudan. The second time period covers a phase in the conflict when the majority of children and young adults returned from the LRA through the reception centres in the wake of the Uganda army’s activities north of the border. The third time period covers the period after the LRA were drawn into peace negotiations. A map of the districts where these individuals were traced is shown in Fig. 1.  An individual found in Kampala and another individual  located in Kenya are not indicated on the map, and the current home was not recorded for four individuals.

**Table 1:** Distribution of records, sample, and individuals traced, by year at GUSCO

Distribution of records and sample	All years	Year at GUSCO		
		1997–2001	2002–2005	2006–2012
All GUSCO records	3040	770	1810	460
Total sample taken from 3040 records	304	77	181	46
Number of females in sample	103	15	63	15
Number of females not found	27	0	16	1
Percent of females traced	74	100	75	93
Number of males in sample	201	62	118	31
Number of males not found	48	8	49	1
Percent of males traced	76	87	59	97

#### Interviews with individuals traced from GUSCO records

Previous and on-going ethnographic fieldwork undertaken by members of the team in northern Uganda informed the design of in-depth, semi-structured interview schedules as well as the interpretation of data. Some of this fieldwork dates back to the 1980s (for example, [53]) and, taken together, it addresses a broad range of issues on health, well-being and justice in the wake of protracted war and conflict (for example, [54–60]). Insights emerging from participant observation fieldwork carried out in GUSCO in 2004, 2005 and 2006 by MP, TA and JA also informed the design of the interviews and interpretation of data emerging from semi-structured interviews.

Interviews were carried out by an experienced team of ten researchers (including MP, TA, JA, DA, JO), all of whom had previous experience of working with people who had been recruited by the LRA. These interviews took place between 2013 and 2016. They involved eliciting socio-demographic information from each person about their age, ethnicity, place of birth, location and duration of abduction. Time was also spent encouraging interviewees to reflect on their time with the LRA, their experiences of life at the reception centres, and the issues they face in their day-to-day lives.

Forty follow-up interviews were carried out, with a view to cross-checking and, in some cases, filling in missing information from the initial interviews. In the instances where individuals were too young (less than 14 years old) to be interviewed, unavailable or deceased, relatives were interviewed instead. This occurred in 65 cases. After each interview, notes were written up in detail electronically and formatted according to protocol. Responses were checked for agreement with the GUSCO records (notably the amount of time spent with the LRA and whether or not they were returned to a family member’s homestead). Any discrepancies were flagged for follow-up.

### Interview coding

The transcripts were open-coded using thematic analysis to identify patterns in the interviews. The research team collaboratively established an initial codebook based on their experiences of working with the study population. To pilot the codebook, three members of the team worked independently to code the interviews. These codes were then refined collaboratively to finalize a list of recurring themes. The coded documents were then reviewed by another member of the team, with the aim of identifying any outstanding gaps and anomalies. Following discussion, the codebook was further refined by the team. All interviews were then recoded using MAXQDA 11 (VERBI Software, 2019) with the revised coding scheme.

Following the interview text analyses, a set of variables were identified for quantification to facilitate further descriptive and quantitative analyses. Members of the research team encoded additional variables related to living situations, social relationships, health, stigma, *cen*, acceptance, and reintegration. These socio-demographic data were extracted by supplemental codes around, for example, access to vocational training, documented health issues, challenges accessing land, experiences of *cen,* accusations of *cen, and* experiences of stigma.

For the purposes of this research, *cen* refers to a malevolent spiritual force, which emanates from those that have witnessed or perpetrated violence, or been in physical contact with a dead body. *Cen* is manifested in nightmares, interrupted sleep, and disturbed recollections, which may lead to feeling overwhelmed [59]. A given interviewee was coded as “1” if s/he reported having experienced *cen*. If the subject was not raised by an interviewee, then s/he was explicitly asked if they had experienced *cen*. If a given interviewee described being accused of having *cen*, unless s/he felt that they had experienced *cen,* this was recorded as “0” (no *cen*). However, if it was the case that an interviewee was accused of *cen,* whether or not they themselves felt they had experienced *cen*, it was recorded in a separate column that they had been accused of *cen.*

Stigma is a broader term, commonly articulated by calling returning recruits ‘rebel’, ‘killer’, ‘mentally disturbed’ and ‘evil one’. Although there is no single Acholi word which translates as stigma, reported experiences were categorised and coded under this broad term if relatives, neighbours and/or other people within the wider community articulated thoughts which left them feeling singled out, ostracised and unwelcome in their home, school or wider community. A given interviewee was coded as “1” (having experienced stigma) if they themselves described a situation where they had experienced stigma. In many of these cases, this involved the accusation of bringing *cen back from the bush*. Where it was unclear to the interviewer, interviewees were explicitly asked if they had experienced *‘cimu tok’* (finger pointing) which is the most commonly used Acholi phrase to describe stigma. If a given interviewee did not report experiencing any kind of stigma, they were coded as “0” (no stigma).

As with the concepts of *cen* and stigma above, the other variables identified for quantification were encoded systematically using variable definitions developed from the text analyses (see Supplementary Materials for all variable definitions). In the majority of cases, missing data in the quantified variables were attributable to situations where an interviewee felt uncomfortable responding to a specific question and the interviewer recognised that it was inappropriate to press for an answer. There were also circumstances where the interviewee did not know the answer to the question. This was particularly the case where the person being interviewed was a carer or relative rather than a former recruit. All results were exported in a spreadsheet format for subsequent analyses in statistical software packages.

### Analyses of quantified data

Data from the qualitative interviews were merged with data from the original GUSCO records. Descriptive analyses of all variables were conducted to assess the distributions of the data. The distributions of particular variables of interest (notably age, gender, education, living situation) were calculated across various levels of disaggregation. Where relevant, the distributions between genders were compared using t-tests, with significant differences determined at *p*-value <.05. Informed by the results of the interview text analysis, the relationship between an interviewee’s access to land through family and temporal variables capturing their length of time with the LRA, period of time at GUSCO, and length of time since their return were each evaluated using univariate logistic regression (see Supplementary Materials for more information). Logistic regression was also utilised to determine the probability of experiencing *cen* and stigma based on a suite of potential individual-level characteristics, extracted from the individual interviews. Variables with significant relationships with the dependent variable or the potential for significant interactions with other covariates were then included in multivariate analyses to assess covariant significance. Univariate and logistic regressions have been previously used to analyse similar sample sizes for similar exploratory and descriptive purposes (for example, [61–63]). The results were expressed through predicted probabilities of the outcomes of interest. All analyses were conducted in Stata (Version 14) and R (Version 4.0.2.)

### Ethics

In line with UK and Ugandan ethical requirements, the study was explained verbally in Acholi and each participant received a copy of the consent form. Participants provided written informed consent, and in cases of limited literacy, a thumb print. They were usually interviewed, without remuneration, in or close to their homestead. In a small number of cases, participants travelled to an agreed meeting place and their expenses were reimbursed. From the outset, we were aware that some participants might wish to contribute to the research, but also find talking about past and/or current experiences, distressing. In such instances, s/he was asked if they wished to continue with the conversation and reminded, in the nicest possible way, that they should not feel under any pressure to continue. The interviewer(s) were also encouraged to use their own judgement i.e. we were acutely aware of the possibility that, irrespective of any reassurances provided, there might be instances in which the person concerned might feel (incorrectly) under pressure to continue and unable to say what they really felt or thought. Above all, we were guided by the ethical guidelines provided by the Association of Social Anthropology [64], which make clear that primary responsibility is to the welfare of participants, and to ensuring that difficult situations are not made worse.

The availability of psychosocial support is limited in northern Uganda, but in those instances when participants articulated acute distress and suffering, interviewers provided details of a practising psychiatrist at Lacor Hospital, Gulu who was willing to see her/him free of charge. There were a few cases where advice was sought and, where appropriate, travel expenses were covered by the project.

## Results

### Demographic characteristics of the sample

As reported above, a total of 230 individuals were traced from the selected sample of GUSCO records. Of these, 209 were abducted by the LRA and 21 individuals were born of war, that is, their fathers were LRA commanders and they came to the reception centre as babies or young children. Given the distinct experiences between these two groups, their long-term experiences since leaving GUSCO were analysed separately. The results from the 21 individuals who were born of war are presented in the final part of the results section.

With respect to the 209 formerly recruited persons, Table 2 provides an overview of selected demographic characteristics. As shown in the table, the proportion of women and men in our sample approximates to the gender distribution found across all of the reception records (just under one-third women, just over two-thirds men) [23]. The average ages of both the women and men sampled were in their late-20s at the time of interview, and the majority resided in rural areas (approximately 9 out of 10 individuals across both genders.) While approximately the same proportion of men and women completed primary school (around one-third), significantly more men went on to complete secondary school (26%) compared to women (5%). The average time spent with the LRA was also significantly different, with women spending more than twice as many years with the LRA than the men.

**Table 2:** Demographic characteristics of GUSCO sample (excluding children born of war), by gender and total sample

Sample characteristics	Male	Female	Total sample
Number interviewed	144	65	209
Average age (years)	26	27	26
Minimum age (years)	14	18	14
Maximum age (years)	40	43	43
Percent living in rural area	96	86	93
Percent completed primary school	31	34	32
Percent completed secondary school	26*	5*	19
Average time spent with LRA (years)	1.5*	3.5*	2.25
Minimum time spent with LRA (years)	.003	.003	.003
Maximum time spent with LRA	9	13	13
Percent at GUSCO 1997–2001	36	22	32
Percent at GUSCO 2002–2005	47	63%	52
Percent at GUSCO 2006–2012	17	15%	16

Note: * indicates a statistically significant difference between males and females as determined by t-test with *p*-value<.05

**Fig. 2:**
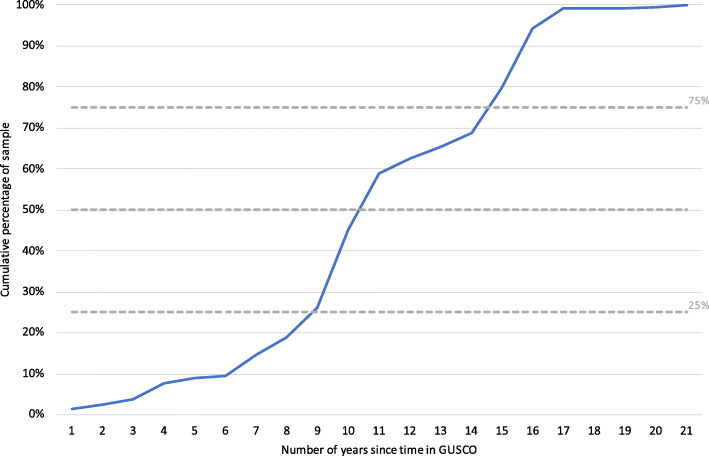
Cumulative percentage of interviewee sample by the number of years since return from GUSCO and the date of the interview

Most interviewees had returned from the LRA several years prior to being interviewed (Fig. 2.) Approximately 90 % of the sample returned 6 or more years prior to interview, 75% returned 9 or more years before the interview, and 50% returned more than 10 years before the interview.

### Social and economic dimensions of return

#### Education and skills training

Interviews revealed that after a period of time at the reception centre, 29% of women and 51% of men returned to formal schooling (Table 3). Approximately one-quarter of these women went on to complete primary school but they did not proceed to secondary school, while over two-thirds did not complete their primary education. Only one woman went on to obtain a lower secondary school certificate (S4). By contrast, 40% of the men who returned to formal schooling completed primary school or completed public examinations in secondary education (S4), while less than half dropped out. Four individuals reported that they were still enrolled in formal education, including one person who reported that he had registered for university. The individuals interviewed who returned to formal education, but did not stay, cited the inability to pay school fees as the primary reason for not continuing with their education.

**Table 3:** Social and economic characteristics by gender

Characteristic	Females	Males
**Education**		
Percent returned to formal schooling (n)	29 (19)	51 (75)
Percent dropped out before completing primary school	68	49
Percent still enrolled in formal education	0	5
Percent completed primary school	26	15
Percent completed secondary school	5	24
**GUSCO Skills Training**		
Percent received any training (n)	34 (22)	22 (32)
Percent still using skills	23	16
**Eking out a living**		
Percent cultivating subsistence crops (n)	93 (52)	90 (118)
Of those cultivating crops, percent participating in other work	60	47
Of those cultivating crops, percent enrolled in full-time education	0	0
Percent not cultivating subsistence crops (n)	7 (4)	10 (15)
Of those not cultivating crops, percent participating in other work	25	67
Of those not cultivating crops, percent enrolled in full-time education	0	33

GUSCO offered some skills training at various points during and following the war. Fifty-four individuals reported that they had received at least one type of training: 34% of women and 22% of men (Table 3). The type of training received included: baking and catering, carpentry, driving and mechanics, bricklaying and construction, tailoring and crafts, and teaching. It is striking that of those individuals who received training, only about one-quarter of the women and less than one-fifth of the men said they were still using these skills. For those who were not utilizing their skills, it was reported that this was because they were redundant in their villages.

#### Eking out a living

Most women and men interviewed relied on subsistence agriculture to survive. Among the women for whom information about subsistence agriculture was available, 93**%** stated that they were cultivating subsistence crops (Table 3). Among the women who did not report cultivating crops, only one reported that she had another job (baking chapatti). Most of the women who reported cultivating subsistence crops also stated that they were participating in additional work. The most prevalent activities among these women were tailoring, selling crops, and brewing alcohol. With respect to the women who cultivated subsistence crops and did not have additional work, our data suggest that most interviewees resided in rural areas (92%), lived in Gulu District (64%), accessed land through their male partner/husband or family (93%), and they all came back through GUSCO between 1997 and 2005.

Similar trends occurred among the men who spoke about subsistence agriculture, with the vast majority (90%) reporting that they were cultivating crops (Table 3). For those not cultivating crops, most reported that they were in full time education or engaged in salaried work (such as working for a hotel, a security organisation**,** the army). Some said that they were also engaged in non-salaried activities, including *boda boda* (motorbike taxi) driving and fishing. Approximately half of the men involved in subsistence agriculture also mentioned that they were doing other work, such as selling crops, *boda boda* driving and construction.

#### Access to land

**Fig. 3:**
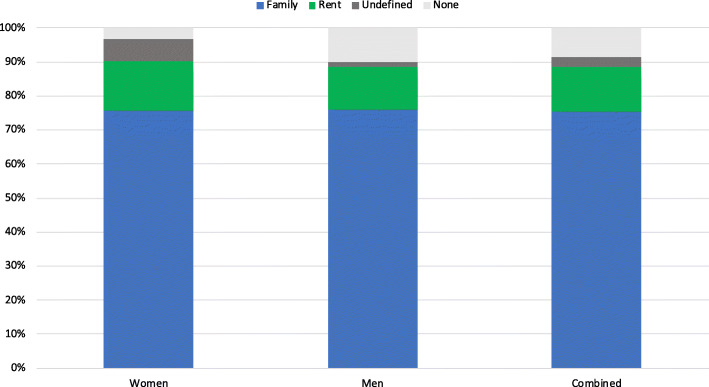
Access to land by gender and combined. Note: For women, access through partners/husbands were considered access through family

Figure 3 presents data showing how access to land varied by gender. Of the women for whom information was elicited, 3% had no access to land and 15% rented land from a landowner. Seventy-six per cent of women accessed family land, but these arrangements were typically complicated and fluid. For women returning with children born of war, access was affected by whether or not her father and brothers were willing to accept her children into the home. It was also contingent on current sexual partners. In many cases, women moved from one partner to another, each time gaining access to their partner’s ancestral land. While such arrangements enabled them to grow food for themselves and their children, they were also spoken about as rather precarious arrangements. The proportion of men accessing land was a bit less (90%) among the men for whom data was available. They either rented land (12%) away from their father’s home or they accessed their ancestral land (75%).

Informed by the results of the interview text analysis, we evaluated how access to land varied in relation to year of return through GUSCO and the length of time spent with the LRA. Interestingly, the year of return through GUSCO was not significantly correlated with whether individuals had access to family land, either as a continuous variable or grouped by the cohorts related to the phase of conflict. However, the length of time spent with the LRA was significantly negatively correlated with access to family land, with increases in the number of months predictive of a lower probability of access to land through the family (*p* < .01) (see Supplementary Materials for full results).

There are several possible inter-related reasons for why access to ancestral land declined so markedly among men and women who had spent many years with the LRA. Under ‘ordinary’ circumstances, Acholi women are able to utilise land without charge, if it belongs to their father, brother or male partner/husband. However, women returning from life with the LRA sometimes ended up feeling so unwelcome that they left their parental or marital home and tried to eke out a living by renting small plots of land, typically on the fringes of small towns such as Gulu. In contrast to women, men have a right to some of the ancestral land of their father’s home area. However, they were returning to a situation in which there was not only a shortage of fertile land and widespread economic impoverishment, but also a strong likelihood that their family circumstances had changed and fear that they were polluted by *cen*. There were numerous cases, for example, when a young man came back from the LRA, only to find that one or both parents had died, or that his parents had separated and/or his father had acquired a new wife. In such circumstances, it was not unusual for his father or father’s brother(s) to dismiss any claims the young man might have to ancestral land and to make life so unpleasant that he ended up moving elsewhere.

#### Intimate relationships

**Fig. 4:**
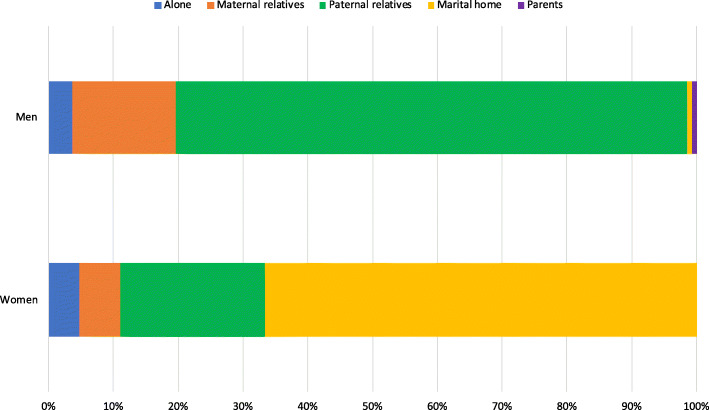
Distribution of who people lived with at the time of interview, by gender

The living situations among the men and women interviewed were often stated to be in flux. At the time of interview, the majority of women either lived with a ‘husband’ or their parent(s), while the majority of men either lived with their in-laws (which indicates a spouse) or their extended paternal family (Fig. 4). However, this figure does not come close to capturing the complexity of issues facing returning women and men. Although it is hard to quantify the quality of relationships, the majority of women reported that they had not had a stable relationship since their time at GUSCO, and only two stated that they had completed the traditional marriage process. More than half also expressed unhappiness with current relationships, or their most recent relationship, citing verbal or physical abuse by their partner, co-wives or in-laws as regular occurrences. Relationships with male partners/husbands typically became fraught following the birth of a child – not least because it triggered conflicts over resources with co-wives and others in the home.

Accusations were frequently made that they must have been involved in murderous acts to have survived their time with the LRA, and that *cen* would create domestic havoc. Women in this situation often ended up leaving their partner’s home and taking their children to their father’s or brother’s homestead. They would often leave them there so that they could ‘move on’ and start co-habiting with another man elsewhere.

The situation with men was rather different. With few exceptions (just four), their time with the LRA did not overtly influence the kind of relationships they established with women on their return; and they did not express dissatisfaction with the stability of these relationships. However, there were several cases where they were clearly aware that their girlfriend or wife had been warned by others not to get involved, or to leave the relationship, on the grounds of being ‘a killer’. In all cases investigated the women concerned dismissed such talk as false rumours. This is not to suggest that other relationships were trouble-free. On the contrary, around 10% of men, for example, spontaneously commented on the financial challenges they faced and indicated that this was a source of conflict, with some female partners/wives leaving them as a result. Other difficulties reported included a female partner having an affair and a violent incident in which a returnee’s female partner broke his collar bone.

#### The enduring nature of rejection

The on-going challenges of accessing education and relevant training, eking out a living and sustaining intimate relationships highlights a crucial point: returning ‘home’ was not straightforward for large numbers of returnees. Following an initial welcome, the majority felt rejected by relatives and/or neighbours. Interviewees spoke about *kwero* (rejection), *cimu tok* (pointing at the back of your head) and a strong sense of *pe gi mita* (a person can stay with you, but they don’t want you). The enduring nature of rejection affected all aspects of life. Ordinary activities such as collecting water from a standpipe or walking down a path to cultivate a plot of land were reported as hard to bear, with relatives and neighbours whispering and muttering under their breath comments such as “what does this rebel want from here?” and “we don’t want you here... you are used to killing people.”

**Fig. 5:**
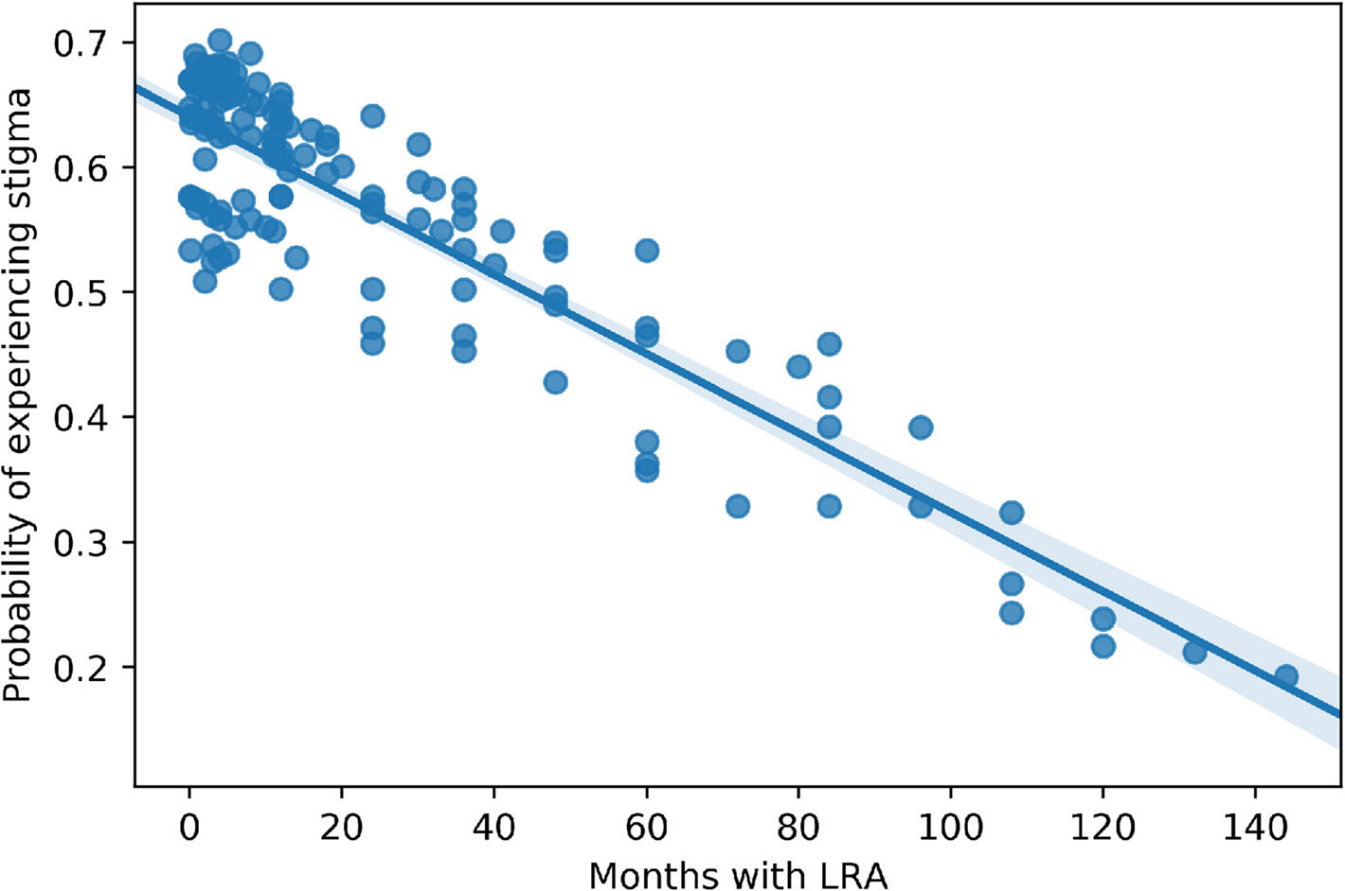
Predicted probability of experiencing stigma by number of months with the LRA

However, experiences of rejection varied. To investigate this empirically, we analysed accounts of verbal or physical incidents conveying stigma. Forty-six percent of women and 59% of men in the sample reported that they had experienced some kind of stigma. To further explore differences amongst those interviewees who experienced stigma and those who did not, the relationships between experiencing stigma since returning from the LRA and several other encoded variables were evaluated using logistic regression. The initial selection of potential correlates was informed by the interview text analyses and previous experiences working with the study population, and included demographic and health characteristics, variables related to their time with the LRA and also those related to their time at GUSCO. The only significant correlation we found through univariate analyses was the length of time (as number of months) spent with the LRA (coefficient = −.011, *p*-value = 0.01.) After accounting for gender and year of return, the number of months spent with the LRA remained a significant predictor (coefficient = −.012, p-value = .03). The predicted probability of experiencing stigma by the number of months spent with the LRA is shown in Fig. 5 (see Supplementary Materials for more details).

The probability of experiencing stigma as predicted by the number of months spent with the LRA was shown to have a negative relationship – that is, the less time spent with the LRA, the more likely individuals were to report that they have experienced stigma. As discussed above, a shorter time spent with the LRA also increased the probability of living on ancestral land. This indicates that experiences of stigma are closely associated with clan relatives and their spouses.

Another factor underlying the rejection of returning recruits is a fear of *cen*. Interviewees spoke of having been affected by *cen,* while living with the LRA and on their return. Twenty-one percent of males and 25% of females mentioned that they had experienced *cen* at some point since their return. In addition, nearly half of the women (49%, *n* = 32) and nearly one-third of the men (32%, *n* = 46) reported that they had been accused of having *cen*, even though they did not think they had been afflicted themselves. Importantly, many of the people with whom returning recruits now lived, feared that *cen* would pass to them. It was also clear that they were frightened by the idea that *cen* could pass from one generation to another, including from a mother to her new-born infant. Accepting sons, daughters and grand-children into the family home thus opened up the possibility of *cen* harming family members that had not been abducted. Both men and women spoke with great feeling about the way in which those in their immediate families, as well as ‘new’ sexual partners, feared that *cen* would harm them too. The following kinds of reported comments were common: ‘“It was written on my back: rebel! Killer! Wife of a murderer!” [and they said that] I was causing diseases within the community, because of the *cen* I came back with’. Similarly, a young woman said that her neighbours had whispered to her ‘new’ partner: “why are you keeping a woman who is possessed by *cen*? One day she will wake up and kill you.**”**

Similar to the analysis exploring factors related to stigma, we sought to evaluate those factors potentially related to experiencing *cen* (see Supplementary Materials for full analysis). Interestingly, there were two factors which were found to be significantly correlated with self-reported *cen*: health problems related to the brain (specifically epilepsy or severe head pains) and not cultivating crops. Using these results, the odds of experiencing *cen* by those not cultivating crops (reference group as those cultivating crops) and those with health problems related to the brain (reference group as those without health problems related to the brain) were calculated. Those reporting health problems related to the brain were nearly seven times as likely to say that they had experienced *cen* (OR = 6.82, *p*-value<.05) compared to those who did not report health problems related to the brain; and those individuals who stated that they were not cultivating crops were nearly three times as likely to say that they had experienced *cen* (OR = 2.897, p-value<.05) compared to those who were cultivating crops.

#### Women with children born to LRA combatants

Many women, struggling to access land and eke out a living, had the additional and related challenge of looking after children whose fathers were LRA combatants. Over one-third of the women in the sample (37%, *n* = 22) reported that they had returned with one or more children born to LRA combatants. Half of these women returned with more than one child, and one woman returned with four children.

Further socio-demographic details about these women are presented in Table 4. Unsurprisingly, the percentage of women who returned with children, and completed primary school, is substantially lower than the sample as a whole (Table 2.)

**Table 4:** Characteristics of women returning with children born to LRA combatants

Sample characteristics	Women returning with children
Number traced	22
Average age (years)	29
Minimum age (years)	18
Maximum age (years)	43
Percent living in rural area	68
Percent completed primary school	18
Percent completed secondary school	5
Average time spent with the LRA (years)	7
Percent at GUSCO 1997–2001	18
Percent at GUSCO 2002–2006	55
Percent at GUSCO 2003–2012	27

Similar to the results shown above on training received at GUSCO, it is striking that while 9 of the 22 women (41%) reported that they had received training in either bakery, catering, or tailoring during their time at the reception centre, only two of them were utilising their skills and these were in tailoring.

On-going challenges facing all women who returned with children include confusion about their lineage status. Historically, bride-price payments clarified their status. Typically, if money was given by the husband’s family to the woman’s family, then the children were deemed to belong to his family. If such payments did not occur, then the children belonged to the mother’s father or brother. A consequence of the protracted war, conflict and economic impoverishment in northern Uganda is that bride-price payments were often not made by those living in IDP camps, and this threw into question the lineage status of the children. The issue is particularly acute for women who returned with children whose fathers were LRA combatants. One young woman, for example, spent 10 years living with the LRA. She returned 7 years prior to the interview with a son. Since her return, she has had another child, but the relationship with this man has not worked out. She said: “I went to my father to find out if I could get some land for farming. [He] just told me that the little piece of land left is to bury him … there is no land and I should look for where the father of my children are.”

Anticipating rejection, this woman made no attempt to contact the child’s paternal relatives. In fact, the majority of women (77%, *n* = 17) have not tried to contact the paternal relatives of their child(ren). They were all aware that concerns would be raised about the lack of bride-price payments, the additional economic demands that would be placed on the family, and widespread fear that their children would pass *cen* to the wider family. In those few cases where contact was made with the child’s paternal relatives, enduring relationships were not established.

In addition to having to cope with the rejection of children by paternal relatives – and in just under a quarter of cases by maternal relatives too - young women have struggled to create a caring environment for their children whilst co-habiting with men. About half of the women who returned with children (*n* = 10) reported that their children had been rejected by their current husband/partner (i.e. the step-father). In the words of one young woman: “when a woman gets married to a man, he will accept you … but when you give birth to their child, he rejects the children you had in the bush.”

Such rejection reflects the fact that their husbands/partners were often under intense pressure from their own relatives to avoid supporting their partner’s other children. One woman, for example, had two children born to LRA combatants and two children with her ‘new’ partner. During the interview, she described how she had spent the morning collecting firewood. When she returned home, she found her mother-in-law putting the two children born to LRA combatants in a basket. Apparently, she could not bear to look at them anymore, and convinced they were possessed by *cen*, planned to ‘throw them away’. The woman felt she had no choice but to pick up her children and move on.

Although the majority of women who returned from the LRA with children articulated a strong sense of rejection (eight reported experiencing stigma and twelve reported accusations or experiences of *cen),* it would be misleading to suggest that they were all surrounded by implacable walls of rejection. More than half of the women (*n* = 12, 55%) reported being able to leave one or more of their children with their mother or brother, while she attempted to start a ‘new’ life with a ‘new’ man. This is not to suggest that the women concerned were happy with such arrangements, as their underlying motive was to protect their children from the experience of being rejected by their ‘new’ partner and/or neighbours, but the support they received should not be under-estimated. There were also situations in which immediate family members made it clear that in spite of having access to a limited amount of fertile land, they would do everything possible to help their daughter/sister, if her endeavour to start a new life accompanied by the children she had had with LRA combatants did not work out.

#### Children born to LRA combatants

Unsurprisingly, the sense of not being welcome and feeling rejected presented on-going and enduring challenges for all 21 children selected from the GUSCO records. Given their young ages, all interviews were conducted with their mothers or grandparents. Thirteen of them lived with their mother, six lived with their maternal grandparent or aunt, and two lived with their paternal grandparents. Of the 16 children eligible to be in school, 13 are still enrolled. An overview of additional demographic characteristics can be found in Table 5.

**Table 5:** Demographic characteristics from sample of children whose fathers were LRA combatants, by gender and total

Sample characteristics	Male	Female	Total sample
Number traced	9	12	21
Average age at time of interview	7	9.25	8.3
Minimum age (years)	3	4	3
Maximum age (years)	12	14	14
Percent living in rural area	92	67	81
Percent of those eligible enrolled at any level of school^a^	60	82	81
Percent of those eligible who dropped out or never enrolled^a^	40	18	19
Average age at GUSCO (years)	2	2.6	2.3
Minimum age at GUSCO (weeks)	32	2	2
Maximum age at GUSCO (years)	3	5	5
Percent at GUSCO 1997–2001	22	17	19
Percent at GUSCO 2002–2005	11	50	33
Percent at GUSCO 2006–2012	67%	33%	48%

^a^Please note that eligibility was determined by those aged 6 years or older

It quickly became evident while interviewing their mothers or other relatives that all the children were having to grapple with a profound sense of rejection. In fact, the caregivers of 15 of the children reported that the child had experienced stigma and 9 had been accused of *cen*. The sentiment displayed in the following comments were common in the interviews: “they don’t like these children. They say they are rebels”; “they say they will grow up to be rebels and disturb them... that is why they don’t accept them in their community.” Rejection is made manifest in multiple ways. It includes: persistent teasing and bullying by schoolchildren; whispering and murmuring by neighbours that the children are unwelcome in their ‘community’; physical violence and neglect by step-fathers and their relatives; rejection by paternal grandparents and related kin; as well as profound ambivalence, if not overt rejection, by maternal grandparents and other close kin. One woman (who spent 10 years with the LRA and returned with a son) described the challenges he now faced in the following way: “He is not in school due to lack of school fees. He is under a hard condition with his grandmother. She does not care about his health … she cooks once a day and this is late at night so in most cases [he] sleeps hungry. She often beats him and tells him that I am possessed by *cen* because I killed many people while in captivity.”

Although the specific challenges facing children vary from case to case, the type of issues mentioned in the quote above affected all but one of the children traced. Inevitably, perhaps, the refusal to pay school fees (even when other children in the same household are being sent to school) combined with a persistent reluctance to share food, provide health care and clothing ends up conveying to the children concerned that they are not at all welcome. Such ambivalence and rejection is, in part, attributable to the ambiguous lineage status of the children concerned.

## Discussion

This article is based on research which has systematically followed up, for the first time, a randomly selected sample of children and young adults who spent time with the LRA, before passing through an internationally-funded reception centre on their way back ‘home’. A minimum of 1 year and a maximum of 21 years had passed since participants first returned from life with the LRA, with 90% having been back 6 or more years at the time of her/his first interview, 75% having been back 9 or more years, and 50% having been back more than 10 years at the time of interview. The research thus provides a useful lens on the longer-term social and economic challenges facing children and young adults returning from a rebel group; and an opportunity to reflect on the ramifications of humanitarian agencies supporting a policy of social re-integration.

Several issues come to the fore, including the following: first, three quarters of those interviewed were found to be living on ancestral land, mostly in very difficult circumstances in rural locations. Importantly, those who spent less time with the LRA were the most likely to be living with their patrilineal relatives, or those of their spouse. They were also the most likely to report incidents which left them feeling singled-out, ostracised and unwelcome in their home or wider community (i.e. stigmatised), and they were the most likely to report being accused of bringing the spiritual affliction *cen* back from the bush.

Second, those young men and women who had been relatively successful adjusting to life back ‘home’ were largely those who had spent the longest amount of time with the LRA. In particular, they were the least likely to report ongoing stigma or *cen.* However, many of them had struggled to adjust to daily life with their clans in rural areas when they first returned and ended up renting land for cultivation and living in urban areas. On-going follow-up research has subsequently revealed that some of them are now networked into support groups such as the Women’s Advocacy Network (WAN) and Watye-Ki-Gen (WKG), which are funded by INGOs and private international donations. These support groups have a tendency to be run by people who acquired considerable authority within the LRA [65]. Although the daily lives of these individuals are far from straightforward, the very characteristics which enabled them to survive and even prosper in the LRA appear to have been an asset following their return [66].

Third, the most vulnerable group were children who were born of war i.e. whose fathers were LRA combatants. This finding corroborates research carried out by other researchers on children born of war in northern Uganda (notably Denov and Lakor [42] and Theidon [48]). However, caution is necessary. On-going research with a much larger sample of 300 women and children suggests trajectories of return for children born of war are profoundly influenced by the status of the child’s mother while she was with the LRA. Children born to senior wives of high-ranking commanders within the LRA, or women who held command positions within the rebel group, appear to be doing better than those children whose mothers were junior wives and/or did not have a rank within the LRA. This is partly because their mothers have re-located to urban and peri-urban areas, but also because LRA hierarchies have been reproduced in peace time and more senior women have been able to access resources from NGOs to pay for primary and secondary education (see the following blogs: [65–67]).

Taken together, these findings both corroborate and diverge from research documenting the realities of return. Numerous scholars, focusing on the immediate and short-term challenges facing returnees have highlighted marginalisation, stigmatisation and social exclusion in northern Uganda (for example, [24, 26, 30]); and elsewhere (for example, [6, 16, 21, 68, 69]). Where research has focused on the longer term issues of return, there is a small amount of evidence to suggest that, with support, some of these issues fade with time. Betancourt’s research [70] on the mental health of ex-child soldiers in Sierra Leone and Boothby et al’s research [19] with male ex-child soldiers in Mozambique both indicate that while former combatants remain troubled and distressed by their experiences many years after their return, these challenges have been mitigated by a variety of interventions. In the case of Sierra Leone, this has included community-wide programmes enabling public authority figures to actively foster processes of social reintegration. In the case of Mozambique, the provision of psychosocial assistance over a period of 6 months at rehabilitation centres, and a commitment to following up on the welfare of children after placing them back home, appears to have helped. However, Boothby’s research [18], in combination with Jordan et al’s research (2012) in Burundi indicates that it is much easier to foster processes of reintegration when skills training programmes, equipment kits, micro-finance support and, ideally, wider structural programmes (such as the rebuilding of hospitals) are made available to non-combatants too. In such contexts, returning combatants are reported to be doing as well – and in the case of Mozambique, slightly better – than peers of a similar socio-economic status who had not perpetrated or witnessed extreme violence and murder as a child soldier.

Boothby et al. [18], Jordans et al. [19] and Betancourt et al’s [70] research, among others, have highlighted the importance of recognising the way in which broader political, economic and social issues shape experiences of return. In northern Uganda, the vast majority of former recruits were returned to internal displacement camps. Here, their relatives and friends were living in extreme poverty, with no or limited access to education, and minimal opportunities for cultivation and salaried employment. Malaria, cholera and other infectious diseases were endemic and biomedical health care limited. The longer term impact of these experiences is not known, but it is likely that they have had a negative and enduring impact on health and well-being, education and subsequent income.

Nevertheless, social reintegration has not occurred in the way anticipated by humanitarian aid agencies promoting DDR. That is particularly so for the vast majority of formally recruited children who continue to feel rejected by former friends, relatives and/or neighbours. There are exceptions. They are for the most part those who were able to secure training and support, in a comparable way to those described by Boothby [17] and Jordans et al. [19]. However, most were sent ‘home’ with just a bit of money and few commodities, but no new skills or fees for secondary school**.** Concerns about what they may have done to survive life with the LRA continue to adversely affect social relationships.

These findings resonate with Akello’s recent research [71] on former LRA combatants, who were reintegrated through the official Ugandan amnesty process (rather than the aid-financed reception centres established for returned children), and live in rural areas. In particular, Akello found that survivors of LRA violence (non-recruits) engage in everyday acts of persecution against returned recruits, with the intention of making the lives of those returned recruits ‘unbearable’ (2019: 264–265).

This insight builds on work in the region that has shown how the scapegoating of vulnerable individuals can become a means of promoting social cohesion and mutuality, especially in highly impoverished post conflict circumstances [72–74].

Many of the ongoing challenges returnees face, for example in relation to livelihoods and settlement patterns, are shared by those who experienced displacement [45, 60]. By most criteria, the livelihoods of those who returned from the LRA are not strikingly different from others in the population that lived in IDP camps. Such challenges are intimately related to high degrees of poverty [75], especially within rural areas, and the broader legacies of conflict [76]. Nevertheless, the findings presented in this article, alongside the work of Akello [71], suggest that those who return from the LRA continue to face additional social challenges. Importantly, familial and community relationships that are crucial for well-being are likely to be unpredictable. In fraught circumstances affecting the whole population, returnees are acutely aware of their vulnerability. They know that tensions can easily escalate into outright rejection, especially in relation to disputes over access to land for cultivation and other resources.

In retrospect, it was inappropriate to assume that the best way to build peace, and to assist children and young adults who had been compelled to perpetrate murder and torture was to quickly place them back ‘home’ with their relatives, many of whom had witnessed violence by the returnees themselves. Time has shown that most young people returning from the LRA have become an underclass, even among the most impoverished, and a considerable focus of fear. Such fear is less about the possibility of impending violence, and more about spiritual and moral pollution. It makes their very presence offensive.

### Limitations

There are several limitations with the findings presented in this article. First, the number of missing records is unknown; and there is no way of knowing whether or not these records concern individuals who had had experiences with the LRA which were systematically different from those people who were successfully traced. Given the poor state of the records which were recovered, and the haphazard way in which they had been stored, it seems unlikely, however, that a random sample was not selected.

A second related limitation concerns the challenges of interpreting data from the sample selected. While 76% of individuals were successfully traced and interviewed, it was not possible to trace the remaining 24%, and there is no way of knowing whether this latter group were systematically different from those who were traced and interviewed. Nevertheless, the data presented in this article has provided an opportunity to enlist the participation of people who may otherwise have been missed because they were not living in urban or peri-urban areas and difficult to locate. In so doing, it was possible to look more closely at the longer term social realities of promoting return and reintegration for a wider range of people in a way that smaller samples, based on snowball sampling or links with NGOs, have not been able to do.

A third limitation concerns the interviews themselves. By any standard, the response rate was high and some readers may be concerned that this reflects unfortunate power dynamics, whereby potential respondents did not feel able to refuse an interview. While it cannot be proven that this was not the case, it is important to note that former GUSCO staff were actively involved in tracking down and talking to former child recruits. They were the first point of contact and the high response rate almost certainly reflects the quality of relationships they had established with them at the reception centre. In addition, all researchers had extensive knowledge of the region and people, and considerable experience of talking about complex and sensitive issues concerning social reintegration. They had received considerable training in discussing difficult and emotional issues with those affected by war and conflict and/or were trained in anthropology (which places great emphasis on asking open-ended questions, avoiding judgement and leading questions etc).

Although the researchers carrying out the interviews had extensive knowledge of the region and people, and considerable experience of talking about complex and sensitive issues concerning social reintegration, it was not always possible to establish sufficient trust and rapport during interviews to elicit reliable information. In circumstances where a researcher felt information was missing, incomplete or differed markedly from the original GUSCO records, follow-up interviews were arranged. Nevertheless, it is possible that some experiences went undetected.

A fourth limitation concerns the challenges of defining *cen* and stigma. As noted previously, *cen* and stigma have varying interpretations. In this article, emphasis is given to the way in which people talk about *cen* and stigma in relation to the reintegration of children returning from life with the LRA. Regarding *cen* in particular, in this context we recognise this ascribes a malevolence to *cen* and that there may be an overlap with other spiritual forces like *tipu* and *jok* within this definition.

## Conclusion

The research findings presented in this article suggest that returning children and young adults are not a homogenous group. Experiences are not only profoundly shaped by gender and age, but also by the amount of time spent with the LRA and place of residence. It was particularly striking that those men and women who spent the least amount of time with the LRA now usually live in rural areas, where they are more likely to report experiences indicating on-going rejection, hostility and stigma than those who had spent a longer period of time with the LRA, and who are commonly not now living on patrilineal land. This calls into question the merits of continuing to assume that it is necessarily appropriate to place returning children and young adults back with their families when they return from war and conflict, particularly where facilities and funding are not in place for systematic follow up and monitoring. The fact that follow up and monitoring was not put in place, and not prioritised, is also troubling. International humanitarian aid agencies actively supported the work of GUSCO, and other reception centres in northern Uganda. Working closely with the Ugandan military and other government officials, they provided the necessary resources to enable returning recruits to be placed back ‘home’ even as the fighting, abductions and forced displacement continued. Then, when the war ended, the agencies moved on to other projects, and the returned children left to their own devices. Apart from the GUSCO records salvaged by the research team, most of their registration documents were lost or destroyed. The enduring legacies of this neglect are highlighted in this article.

## Supplementary Information

**Additional file 1: Table S1.** Definitions of variables quantified from interview texts. **Table S2.** Evaluation of relationship of temporal variables to access to ancestral land. **Figure S1.** Predicted probability of access to ancestral land over months spent with the LRA. **Table S3.** Predictors of stigma, univariate logistic regression results. **Table S4.** Parameter estimates, multivariate regression results. **Table S4.** Potential determinants of *cen*, univariate logistic regression results.

## Data Availability

The data informing this article are not publicly available due to the importance of maintaining confidentiality and anonymity of study participants. Excerpts from the interviews illustrating important points in this article may be requested from the corresponding author.

## Abbreviations

DRC: Democratic Republic of the Congo
GUSCO: Gulu Support the Children Orgaisation
LRA: Lord’s Resistance Army
UPDF: Uganda People’s Defence Force
