# Supplementary material for: Legacies of humanitarian neglect: long term experiences of children who returned from the Lord’s Resistance Army in Uganda
Source: Confl Health. 2021 May 29;15:43. doi: 10.1186/s13031-021-00374-5 (PMC8164804; doi:10.1186/s13031-021-00374-5)
Supplement: Supplementary file 1 — Additional file 1: Table S1. Definitions of variables quantified from interview texts. Table S2. Evaluation of relationship of temporal variables to access to ancestral land. Figure S1. Predicted probability of access to ancestral land over months spent with the LRA. Table S3. Predictors of stigma, univariate logistic regression results. Table S4. Parameter estimates, multivariate regression results. Table S4. Potential determinants of cen, univariate logistic regression results. [file 13031_2021_374_MOESM1_ESM.docx]

**Legacies of the Lord’s Resistance Army: a follow up study of former child recruits**

***Supplementary materials***

**Melissa Parker,^1^ Cristin A. Fergus,^2^ Charlotte Brown,^2^ Dorothy Atim,^3^ James Ocitti, ^3^ Jackline Atingo^3^ and Tim Allen^2^**

**^1^ Department of Global Health and Development, London School of Hygiene and Tropical Medicine, ^2^ Firoz Lalji Centre for Africa, London School of Economics, ^3^Firoz Lalji Centre for Africa (Uganda)**

Contents

[A. Quantification of variables from interview texts 2](#_Toc57114076)

[B. Evaluation of relationships between temporal variables and access to ancestral land 4](#_Toc57114077)

[C. Evaluation of relationships between experiencing stigma and interviewee characteristics 5](#_Toc57114078)

[D. Determinants of experiencing cen 6](#_Toc57114079)

# **A. Quantification of variables from interview texts**

Table S1 shows the variables quantified from the interview texts, the definitions used to extract the information, and whether they were included in the subsequent analyses.

Table S1. Definitions of variables quantified from interview texts

| **Variable** | **Interview text encoding process definition** | **Number of interviewees with this information^1^** | **Variable included in ancestral land access analysis** | **Variable included in stigma analysis** | **Variable included in *cen* analysis** |
| --- | --- | --- | --- | --- | --- |
| Age (years) | Age in years of individual as reported by interviewee at the time of the first interview and confirmed with GUSCO records | 199 | no | yes | yes |
| Time with the LRA (months) | Total number of months spent with the LRA reported by interviewee and calculated from GUSCO records | 190 | yes | yes | yes |
| Number of years since GUSCO | Number of years since the individual’s time at GUSCO calculated from year of return noted in GUSCO file and recorded at time of first interview | 191 | no | yes | yes |
| Year at GUSCO | Year the individual arrived at GUSCO obtained from records | 191 | yes | yes | yes |
| Number of abductions | Total number of abductions by the LRA as reported by the interviewee | 199 | no | yes | yes |
| Abducted by the LRA | The individual considered that they had been abducted by the LRA (as opposed to joining voluntarily) and this was confirmed from the GUSCO records | 199 | no | yes | yes |
| Resettlement location | The location the individual went to when they left GUSCO obtained from records | 209 | no | no | no |
| Education | Level of formal education reported by the individual at the time of the interview | 117 | no | no | no |
| Still in education | The interviewee reported that they were still in education at the point of the interview | 204 | no | no | no |
| Lives with parent(s) | Interviewee reported that they lived with one or both of their parents at the time of the interview | 209 | no | yes | yes |
| Lives with maternal relatives | Interviewee reported that they lived with maternal relatives at the time of the interview | 209 | no | yes | yes |
| Lives with paternal relatives | Interviewee reported that they lived with paternal relatives at the time of the interview | 209 | no | yes | yes |
| Lives with spouse | Interviewee reported that they lived with spouse at the time of the interview | 209 | no | yes | yes |
| Lives with in-laws | Interviewee reported that they lived with their in-laws at the time of the interview (but not with the spouse present) | 209 | no | yes | yes |
| Eking out a living | The individual reported that at the time of the interview they were engaging in no work or irregular, non-salaried work and cultivating subsistence crops | 209 | no | yes | yes |
| Cultivating crops | The individual reported that at the time of the interview they were cultivating subsistence crops | 189 | no | yes | yes |
| Any access to land | The individual reported any access to land at the time of the interview | 200 | no | yes | yes |
| Land access through spouse | The individual reported that they had access to land through their spouse at the time of the interview | 200 | no | yes | yes |
| Land access through family | The individual reported that they had access to land through their family (parents or extended) at the time of the interview | 200 | no | yes | yes |
| Land access through renting | The individual reported that they had access to land through rented plots at the time of the interview | 200 | no | yes | yes |
| Vocational training | The individual reported that they had received vocational training at some point since their return from the bush | 54 | no | no | no |
| Training type | The type of vocational training the individual received (e.g. tailoring, construction) | 54 | no | no | no |
| Training use | The individual reported that they are, at the time of the interview, they are still using the vocational training they received | 54 | no | no | no |
| Current partner is from the LRA | The individual reported that their current partner is the same partner they had during their time in the bush | 46 | no | yes | yes |
| Reported sexual violence since return | The individual reported any sexual violence since their time at GUSCO at the point of interview | 65 | no | yes | yes |
| Any reported health problems | The individual reported any current health problems at the time of the interview | 208 | no | yes | yes |
| Health problem: chest pains | The individual reported current or ongoing chest pains at the time of the interview | 128 | no | yes | yes |
| Health problem: abdomen pains | The individual reported current or ongoing abdominal pains at the time of the interview | 107 | no | yes | yes |
| Health problem: limb pains | The individual reported current or ongoing limb pains at the time of time of the interview | 103 | no | yes | yes |
| Health problem: infectious disease | The individual reported current or ongoing health issues related to an infectious disease at the time of the interview | 98 | no | yes | yes |
| Health problem: brain-related issues | The individual reported current or ongoing health difficulties related to the brain, e.g. seizures at the point of the interview | 88 | no | yes | yes |
| Rural location | The individual lived in a rural location at time of interview as determined by the interviewer’s notes and descriptions | 209 | no | no | no |
| Partner is also a formerly abducted person | The individual reported that their current partner (at the point of interview) is also a formerly abducted person but not the same partner they had during their time in the bush | 46 | no | no | no |
| Formal school return | The individual reported that they went to formal schooling upon their return from GUSCO | 94 | no | no | no |
| Additional work | At the point of interview, the individual reported that separate to subsistence cultivation they engage in some form of paid work - this could include non-salaried work as well as salaried work. The type of work was noted (e.g. brickmaking, selling crops, security official) | 145 | no | no | no |
| Traditional marriage process | The individual reported at the point of interview that they had completed the traditional Acholi marriage process at some point since leaving GUSCO | 202 | no | no | no |
| Dissatisfaction with current or most recent relationship | The individual reported at the point of interview that they had experienced verbal or physical abuse by partner, co-wives or in-laws in their current or most recent relationship | 202 | no | no | no |
| Relationship | The individual reported at the point of interview that they were cohabiting with a partner or in some other form of relationship | 202 | no | no | no |
| Cen^2^ | The individual reported at the point of the interview whether they had experienced *cen^2^* | 185 | no | no | Outcome variable |
| Stigma^3^ | The individual reported at the point of the interview whether they had experienced stigma | 184 | no | Outcome variable | no |

^1^This column refers only to the adults interviewed and does not include children born to LRA combatants

^2^ The term *cen* refers to a malevolent spiritual force, which emanates from those that have witnessed or perpetrated violence, or been in physical contact with a dead body. *Cen* is manifested in nightmares, interrupted sleep, and disturbed recollections, which may lead to feeling overwhelmed. A given interviewee was coded as “1” if s/he reported having experienced *cen*. If the subject was not raised by an interviewee, then s/he was explicitly asked if they had experienced *cen*. If a given interviewee described being accused of having *cen*, unless s/he felt that they had experienced *cen,* this was recorded as “0” (no *cen*). However, if it was the case that an interviewee was accused of *cen,* whether or not they themselves felt they had experienced *cen*, it was recorded in a separate column that they had been accused of *cen.*

^3^ Stigma is commonly articulated by calling returning recruits ‘rebel’, ‘killer’, ‘mentally disturbed’ and ‘evil one’. Although there is no single Acholi word which translates as stigma, reported experiences were categorised and coded under this broad term if relatives, neighbours and/or other people within the wider community articulated thoughts which left them feeling singled out, ostracised and unwelcome in their home, school or wider community. A given interviewee was coded as “1” (having experienced stigma) if they themselves described a situation where they had experienced stigma. In many of these cases, this involved the accusation of bringing *cen back from the bush*. Where it was unclear to the interviewer, interviewees were explicitly asked if they had experienced *‘cimu tok’* (finger pointing) which is the most commonly used Acholi phrase to describe stigma. If a given interviewee did not report experiencing any kind of stigma, they were coded as “0” (no stigma).

# **B. Evaluation of relationships between temporal variables and access to ancestral land**

Table S2 shows the potential temporal determinants of access to ancestral land by the individuals in the sample (see Table S1 for the variables included in the analysis.) The categorical cohort variables were assigned as dummy variables from the continuous “Year at GUSCO obtained from records” variable. The three cohorts represent different phases of the conflict: 1997-2001, 2002-2005, and 2006-2016. The first time period refers to the period before the Ugandan army began attacking LRA bases in South Sudan. The second time period covers a phase in the conflict when the majority of children and young adults returned from the LRA through the reception centres in the wake of the Uganda army’s activities north of the border. The third time period covers the period after the LRA were drawn into peace negotiations. Figure S1 shows the predicted probability of access to ancestral land using the logistic regression results from this analysis of the time with the LRA in months.

Table S2. Evaluation of relationship of temporal variables to access to ancestral land

| **Potential temporal relationship to ancestral land access** | **Coeff.**  **(log odds)** | *Std error* |
| --- | --- | --- |
| Year at GUSCO | -0.148 | *0.125* |
| Arrived at GUSCO during phases of the war: |  |  |
| Cohort 1 arrived between 1997-2001 | 0.608^+^ | *0.313* |
| Cohort 2 arrived between 2002-2005 | -0.287 | *0.282* |
| Cohort 3 arrived between 2006-2012 | -0.405 | *0.376* |
| Time with the LRA (months) | -0.013** | *0.005* |
| Number of years since GUSCO | 0.059 | *0.042* |

Note: Logistic regression coefficients with standard errors; ** p<0.01, * p<0.05, + p<0.1. The constants were estimated but not shown. Dependent variable is reported access

ancestral land at the time of the interview.

Figure S1. Predicted probability of access to ancestral land over months spent with the LRA

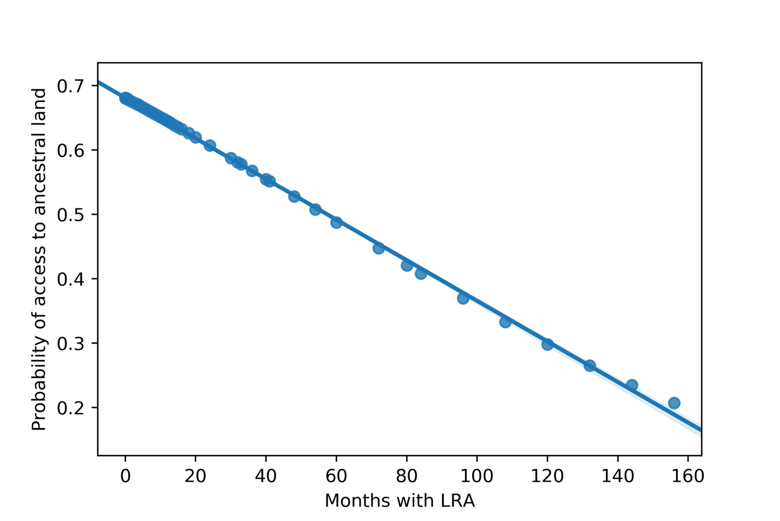


**C. Evaluation of relationships between experiencing stigma and interviewee characteristics**

The variables included in the analysis on experiencing stigma are shown in Table S1. Table S3 shows the regression coefficients and standard errors for the univariate logistic regression analyses.

Table S3. Predictors of stigma, univariate logistic regression results

| **Potential determinants of stigma** | **Coeff.**  **(log odds)** | *Std error* |
| --- | --- | --- |
| Age (years) | -0.007 | *0.028* |
| Time with the LRA (months) | -0.011** | *0.005* |
| Number of years since GUSCO | -0.017 | *0.041* |
| Year at GUSCO | -0.015 | *0.038* |
| Number of abductions | -0.224 | *0.274* |
| Female | -0.531^+^ | *0.301* |
| Method of joining LRA (reported abducted) | -0.206 | *0.382* |
| No formal education | -1.357^+^ | *0.829* |
| At least some primary education | 0.411 | *0.304* |
| At least some secondary education | 0.501 | *0.365* |
| Lives with parent(s) | 0.147 | *0.328* |
| Lives with maternal relatives | -0.219 | *0.721* |
| Lives with paternal relatives | 0.210 | *0.331* |
| Lives with spouse | -0.035 | *0.341* |
| Lives with in-laws | -0.340 | *0.461* |
| Eking out a living | 0.779 | *0.505* |
| Cultivating crops | 0.438 | *0.485* |
| Any access to land | 0.785 | *0.506* |
| Land access through spouse | -0.325 | *0.401* |
| Land access through family | -0.617 | *0.514* |
| Land access through renting | -0.240 | *0.419* |
| Experienced or accused of cen | -0.171 | *0.347* |
| Current partner is from the LRA | 1.163^+^ | *0.679* |
| Reported sexual violence since return | -0.904^+^ | *0.523* |
| Any reported health problems | -0.560^+^ | *0.287* |
| Health problem: chest pains | -0.460 | *0.386* |
| Health problem: abdomen pains | -0.241 | *0.515* |
| Health problem: limb pains | -0.426 | *0.563* |
| Health problem: infectious disease | -0.154 | *0.682* |
| Health problem: brain-related issues | -0.965 | *0.939* |

Note: Logistic regression coefficients with standard errors; ** p<0.01, * p<0.05, + p<0.1. The constants were estimated but not shown. Dependent variable is reported experience of stigma since returning from the LRA and GUSCO.

Based on the results of the univariate regression analyses and the interview text analyses, the subsequent multivariate analysis included the only statistically significant (p<0.05) independent variable (Time with the LRA) as well as two additional independent variables, whether the interviewee was a female and the number of years since their time at GUSCO. The results are shown in Table S4.

Table S4. Parameter estimates, multivariate regression results

| **Potential determinants of stigma** | **Coeff.**  **(log odds)** | *Std error* |
| --- | --- | --- |
| Time with the LRA (months) | -0.012* | *0.005* |
| Number of years since GUSCO | -0.024 | *0.042* |
| Female | 0.423 | *0.359* |
| Constant | 0.134 | *0.831* |

Note: Logistic regression coefficients with standard errors; ** p<0.01, * p<0.05, + p<0.1. The constants were estimated but not shown. Dependent variable is reported experience of stigma since returning from the LRA and GUSCO.

Based on Table S4, the following resultant equation was used to predict the probability of having experienced stigma:

log(p/1-p) = 0.134 – 0.012*Time with the LRA – 0.025*Number of years since GUSCO + 0.423*Female

*were p is the probability of having experienced stigma*

# **D. Determinants of experiencing *cen***

The variables included in the analysis on experiencing *cen* are shown in Table S1. Table S4 shows the regression coefficients and standard errors for the univariate logistic regression analyses.

Table S4. Potential determinants of *cen*, univariate logistic regression results

| **Potential determinants of cen** | **Coeff.**  **(log odds)** | *Std error* |
| --- | --- | --- |
| Age (years) | 0.014 | *0.034* |
| Time with the LRA (months) | -0.002 | *0.005* |
| Number of years since GUSCO | -0.016 | *0.047* |
| Year at GUSCO | -0.018 | *0.045* |
| Number of abductions | -0.113 | *0.347* |
| Female | 0.120 | *0.365* |
| Method of joining LRA (abducted or not) | 0.067 | *0.462* |
| No formal education | -0.160 | *0.835* |
| At least some primary education | 0.064 | *0.359* |
| At least some secondary education | -1.079 | *0.568* |
| Lives with parent(s) | -0.812 | *0.485* |
| Lives with maternal relatives | 0.459 | *0.751* |
| Lives with paternal relatives | 0.130 | *0.398* |
| Lives with spouse | 0.746 | *0.392* |
| Lives with in-laws | 0.622 | *0.493* |
| Eking out a living | 0.778 | *0.537* |
| Cultivating crops | -1.062* | *0.535* |
| Any access to land | -0.421 | *0.652* |
| Land access through spouse | 0.001 | *0.483* |
| Land access through family | -0.224 | *0.350* |
| Land access through renting | 0.578 | *0.670* |
| Experienced or accused of cen | -0.111 | *0.509* |
| Current partner is from the LRA | -0.906 | *0.867* |
| Reported sexual violence since return | 1.056 | *0.694* |
| Any reported health problems | 0.501 | *0.354* |
| Health problem: chest pains | 0.768 | *0.460* |
| Health problem: abdomen pains | 0.129 | *0.640* |
| Health problem: limb pains | 0.639 | *0.622* |
| Health problem: infectious disease | 0.940 | *0.812* |
| Health problem: brain-related issues | 1.920* | *0.909* |

Note: Logistic regression coefficients with standard errors; ** p<0.01, * p<0.05, + p<0.1. The constants were estimated but not shown. Dependent variable is reported experience of cen since returning from the LRA and GUSCO.
